# Genomic prediction based on selected variants from imputed whole-genome sequence data in Australian sheep populations

**DOI:** 10.1186/s12711-019-0514-2

**Published:** 2019-12-05

**Authors:** Nasir Moghaddar, Majid Khansefid, Julius H. J. van der Werf, Sunduimijid Bolormaa, Naomi Duijvesteijn, Samuel A. Clark, Andrew A. Swan, Hans D. Daetwyler, Iona M. MacLeod

**Affiliations:** 1 Sheep-CRC, Armidale, NSW 2351 Australia; 2 School of Environmental & Rural Science, University of New England, Armidale, NSW 2351 Australia; 3 Agriculture Victoria, AgriBio, Centre for AgriBioscience, Bundoora, VIC 3083 Australia; 4 Animal Genetics and Breeding Unit (AGBU), Armidale, NSW 2351 Australia; 5 School of Applied Systems Biology, La Trobe University, Bundoora, VIC 3083 Australia

## Abstract

**Background:**

Whole-genome sequence (WGS) data could contain information on genetic variants at or in high linkage disequilibrium with causative mutations that underlie the genetic variation of polygenic traits. Thus far, genomic prediction accuracy has shown limited increase when using such information in dairy cattle studies, in which one or few breeds with limited diversity predominate. The objective of our study was to evaluate the accuracy of genomic prediction in a multi-breed Australian sheep population of relatively less related target individuals, when using information on imputed WGS genotypes.

**Methods:**

Between 9626 and 26,657 animals with phenotypes were available for nine economically important sheep production traits and all had WGS imputed genotypes. About 30% of the data were used to discover predictive single nucleotide polymorphism (SNPs) based on a genome-wide association study (GWAS) and the remaining data were used for training and validation of genomic prediction. Prediction accuracy using selected variants from imputed sequence data was compared to that using a standard array of 50k SNP genotypes, thereby comparing genomic best linear prediction (GBLUP) and Bayesian methods (BayesR/BayesRC). Accuracy of genomic prediction was evaluated in two independent populations that were each lowly related to the training set, one being purebred Merino and the other crossbred Border Leicester x Merino sheep.

**Results:**

A substantial improvement in prediction accuracy was observed when selected sequence variants were fitted alongside 50k genotypes as a separate variance component in GBLUP (2GBLUP) or in Bayesian analysis as a separate category of SNPs (BayesRC). From an average accuracy of 0.27 in both validation sets for the 50k array, the average absolute increase in accuracy across traits with 2GBLUP was 0.083 and 0.073 for purebred and crossbred animals, respectively, whereas with BayesRC it was 0.102 and 0.087. The average gain in accuracy was smaller when selected sequence variants were treated in the same category as 50k SNPs. Very little improvement over 50k prediction was observed when using all WGS variants.

**Conclusions:**

Accuracy of genomic prediction in diverse sheep populations increased substantially by using variants selected from whole-genome sequence data based on an independent multi-breed GWAS, when compared to genomic prediction using standard 50K genotypes.

## Background

Accuracy of genomic prediction of genetic merit of farm animals depends on factors such as heritability, the size of the reference population and the effective number of chromosome segments, which in turns depends on the effective population size ($N_{e}$) [1, 2] and the structure of the reference population used for prediction [3]. In multi-breed/crossbred populations, such as in Australian sheep, it is difficult to achieve a sufficiently high genomic prediction accuracy for genetically diverse breeds with large $N_{e}$ or for minor breeds with small reference populations. Prediction based on a set of reference animals from other breeds was shown to be of limited value [4, 5], which could be mainly due to low linkage disequilibrium (LD) between genetic markers used in common marker genotype arrays and quantitative trait loci (QTL) affecting the traits. Low LD suggests that denser marker genotypes could improve genomic prediction accuracy. However, the use of high-density (500k) genotypes was shown to lead to only small improvements in prediction accuracy in multi-breed sheep data, compared to using a 50k SNP array [6]. The relatively small difference may be due to the LD between genetic markers and QTL remaining low when using common HD genotypes, especially across breeds. Whole-genome sequence data provide further opportunities and have the advantage of covering all causal mutations that are responsible for the genetic variation of a polygenic trait or genetic markers in sufficiently high LD with causal mutations.

Genetic variants at or in high LD with causal mutations are likely to be only a small subset of the whole set of sequence variants. This means that a large part of the whole-genome sequence data is in low LD with causal mutations and can be excluded from the prediction model. Earlier work in dairy cattle showed that WGS data used in genomic prediction gave no to limited improvement in prediction accuracy when all variants were used [7–9] and only small improvements were found by using a subset of variants selected based on genome-wide association studies (GWAS) [9–12]. However, using selected variants can also lead to considerable bias, if the data used for detecting QTL was the same as the training data used to predict breeding values [11, 13].

Reasons for the limited improvement from using WGS data in previous studies may be the high levels of LD and relatively low genetic diversity in the dairy cattle populations used in these studies. With high LD, the difference in predictive ability between markers from standard 50k SNP-panels and other markers closer to the actual QTL is likely to be smaller. Indeed, it will be more difficult to distinguish between these when selecting variants from GWAS in such populations. Improvements in prediction accuracy were more notable when variants were selected from a multi-breed GWAS [14].

Another important aspect of genomic prediction is the genetic relatedness between the training set and the target animals for which we want to predict the breeding value. In efficient breeding programs, it is likely that most selection candidates have close relatives in the training set, i.e. animals with both genotype and phenotype information are often available from previous rounds of selection. Prediction from closer relatives is less likely to be improved by markers that are closer to the QTL, and therefore WGS data provides limited benefit. However, prediction of more distant target individuals is also relevant, for example when applying genomic prediction to more diverse populations and populations of multi-breed nature, such as in beef cattle and sheep, or when special reference populations are formed for a limited set of individuals measured for traits that are not widely recorded, such as slaughter traits or feed efficiency. Relatedness between training and target individuals may vary considerably in such cases. Another application of genomic prediction could be for genetic benchmarking in commercial herds or flocks in which information on previous breeding history is limited, and the target individuals may be less related to the reference population. Using markers that are closer to the actual QTL could make genomic prediction more robust and applicable to a wider range of individuals with lower genetic relatedness, including crossbreds.

The objective of our study was to evaluate the accuracy of genomic prediction in a multi-breed Australian sheep population for some key lamb production traits using variants from imputed WGS data. We used selected variants obtained via a multi-breed GWAS in a separate discovery dataset and compared best linear unbiased prediction (BLUP) and a Bayesian method to evaluate the potential change in accuracy and bias of genomic prediction when adding these variants to a standard 50k SNP genotype array.

## Methods

### Animals and experimental design

Phenotypes for key meat and wool production traits were extracted for animals in the “Sheep Cooperative Research Centre” database (“Research data”), which includes the “Information Nucleus Flocks” (INF), “Resource Flocks” (RF) and “the Sheep Genomics Flock” (SGF). INF consisted of eight flocks located across different regions of Australia that are linked to each other by the use of common sires through artificial insemination between 2007 and 2011 [15] and the RF followed the same mating design (2012–2017) but only in two flocks. SGF was a single research flock located in southern New South Wales, Australia, with data collected between 2005 and 2006 [16]. Additional phenotypes for on-farm traits recorded in industry flocks (“Industry data”) were extracted from the Sheep Genetics database (http://www.sheepgenetics.org.au) for flocks with animals with genotypes. Both research and industry datasets were from a multi-breed/crossbred sheep population. Most animals were either purebred Merino or crossbreds of Border Leicester (BL), Poll Dorset (PD) or White Suffolk (WS) rams mated to Merino dams or BL × Merino dams.

**Fig. 1:**
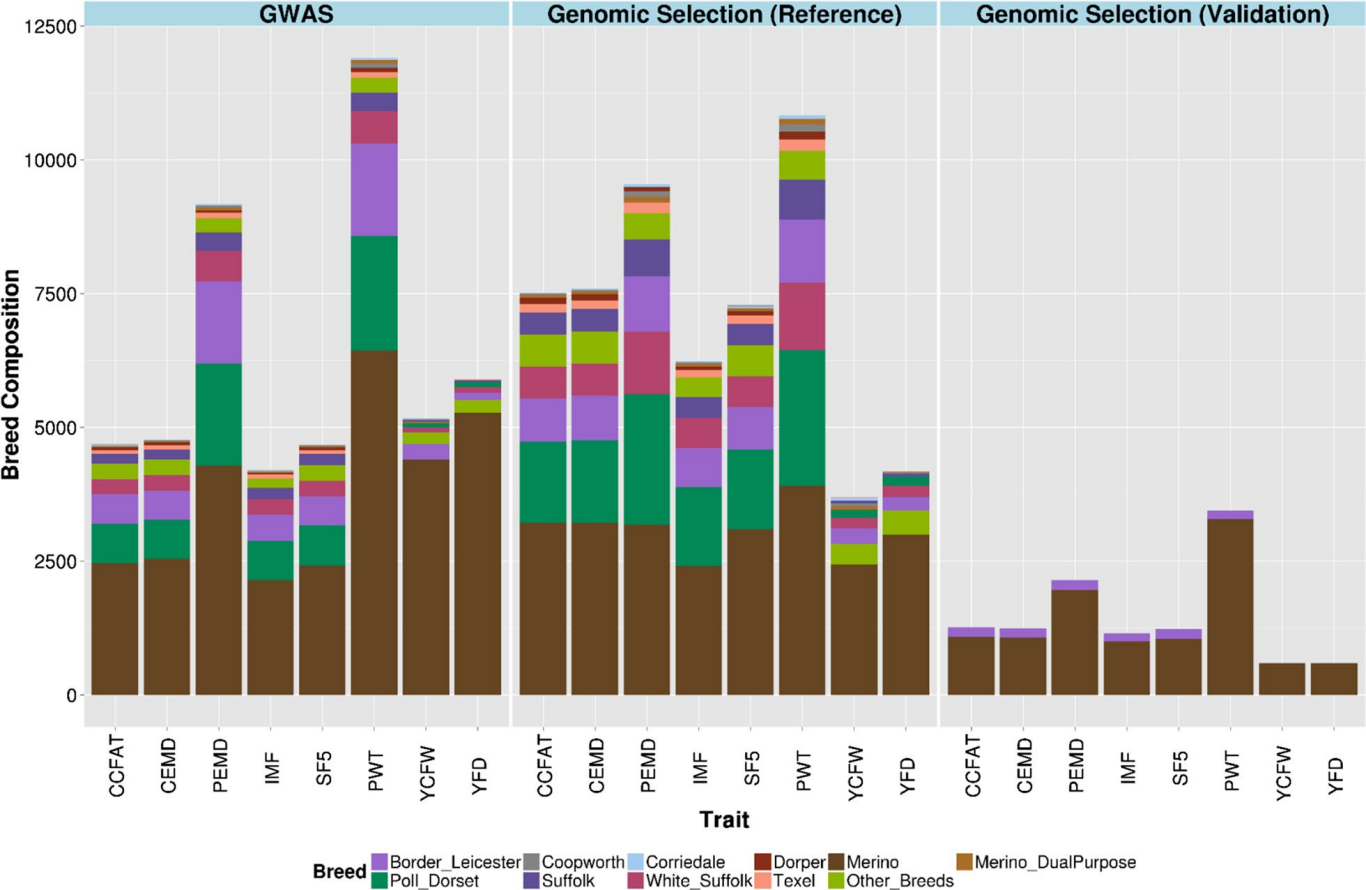
Breed composition of the discovery, reference and validation datasets. Breed composition as number of animals multiplied by breed proportion for each trait in QTL discovery, and genomic selection reference and validation sets

For each trait, animals with both phenotype and genotype data were divided into three non-overlapping groups: (1) a QTL discovery set for a GWAS with imputed WGS data to find variants at or very near putative causal mutations; (2) a reference or training set for genomic prediction based on estimated marker effects; and (3) a validation set to evaluate accuracy and bias of genomic prediction. These three sets were purposefully chosen as being independent of each other to avoid bias in the evaluation of prediction accuracy. The data were divided into three subsets independently for each trait, because the numbers of animals recorded differed for each trait. The QTL discovery and training sets were selected to have a broad representation of breeds and crosses (Fig. 1). However, the validation set consisted of only two specific breed groups: (1) purebred Merino (MER) and (2) F1 cross of Border Leicester with Merino (BL × MER).

The animals in the validation set were selected such that they did not have high genetic relationships with animals in the training set. Thus, for each trait: (1) half-sibs families were not split across the training and validation sets and no sires of validation animals were included in the training set; and (2) relationships between the training and validation sets were further restricted at a fixed threshold using the genomic relationship matrix (GRM) constructed from 50k SNP-chip genotypes [17]. The applied GRM threshold ensured that the genomic relationship between each animal in the validation set with any animal in the training set was lower than 0.25 (meat traits) or lower than 0.125 (wool traits).

The contributions of the MER and BL × MER breeds in the discovery, training and validation sets for each trait are in Table 1 as well as the number of animals from other breeds or crosses available in these three sets for each trait. Proportions of each main breed represented as either purebreds or crosses were predicted using pedigree information (Fig. 1).

**Table 1:** Number of top SNPs and animals of different breeds in different datasets for different traits

Trait (unit)	Number of top SNPs	Discovery (GWAS) set				Reference set				Validation set	
		MER	BL	BL × MER	Other breeds and crosses	MER	BL	BL × MER	Other breeds and crosses	MER	BL × MER
CCFAT (mm)	3989	1157	37	365	3185	761	58	158	6658	912	478
CEMD (mm)	3997	1200	21	389	3215	738	74	161	6741	904	453
PEMD (mm)	3924	2835	860	742	4812	772	216	357	8370	1766	510
IMF (%)	4023	877	11	355	3018	185	25	174	5969	843	415
SF5 (N)	4268	1065	27	358	3291	722	67	143	6460	868	474
PWT (kg)	4287	4691	918	964	5446	1182	262	458	9165	3118	453
YCFW (kg)	3942	3935	41	381	872	1826	73	189	1709	600	–
YFD (µm)	8654	5083	44	62	746	2626	70	135	1432	600	–

Number of top-SNPs and animals of different breeds (*MER* pure Merino, *BL* pure Border Leicester, *BL × MER* crossbred Border Leicester × Merino and other breeds and crosses) in discovery, reference and validation sets for different traits: *CCFAT* carcass fat depth at C site, *CEMD* carcass eye muscle depth, *PEMD* post-weaning eye muscle depth, *IMF* intermuscular fat percentage, *SF5* shear force measured at day 5 after slaughter, *PWT* post-weaning weight, *YCFW* yearling clean fleece weight, *YFD* yearling fiber diameter

### Phenotypes

Phenotypic records of eight traits related to weight, carcass and meat quality and wool production were used in this study; post weaning weight (PWT), post weaning eye muscle depth (PEMD, mm) from ultrasound scanning of live animals, carcass eye muscle depth (CEMD, mm), carcass fat depth at the C site (CCFAT, mm), intra muscular fat (IMF, %) and shear force at 5 days after slaughter (SF5, Newton), yearling clean fleece weight (YCFW, kg) and yearling wool fiber diameter (YFD, µm).

First, phenotypes from the research data were edited for possible outliers (more than 4 standard deviations from the overall mean), for records with no pedigree or breed composition information or for records with missing age or weight at measurement. Phenotypes were then pre-corrected for fixed and random effects in a univariate mixed model analysis, similar to the model used by Sheep Genetics [18]. The fixed effects of the model were a contemporary group effect (from a combination of flock, birth year and management group), gender, age at measurement (days), age of the dam (years; fitted both as a first and second order terms), a combination of birth and rearing type (single, twin, triplet) and traits related to carcass were corrected for weight at measurement. Hence, PEMD was corrected for PWT, whereas CCFAT, CEMD, IMF and SF5 were corrected for carcass weight. For PWT and YCFW a random maternal effect was fitted.

Phenotypes from the industry dataset were taken from the national Sheep Genetics OVIS database [18], which is divided into six separate analyses: Merino, terminal breeds and maternal breeds and smaller runs for Meat Merino, Dohne and Corriedale sheep. These phenotypes were pre-adjusted within each of the six analyses for fixed effects of birth and rearing type, age of measurement in days, age of dam in years with both linear and quadratic adjustments and body weight adjustments for PEMD and carcass traits, as described in [18]. Then, pre-adjusted phenotypes were taken from all data in the national evaluation for each trait and residuals were calculated from fitting a model with a fixed contemporary group effect for PWT and YCFW. These residuals were used as corrected phenotypes for further analysis, but only for animals with genotype information. Animals from the research dataset that were also included in the national evaluation were excluded from the industry dataset. Additional file 1: Table S1 shows the summary statistics of the phenotypes of the combined datasets after editing.

### Genotypes

There were 31,937 animals with genotypes that had either previously been imputed from 12k to the 50k SNP panel (about 35% of animals) or were real 50k genotypes (about 65% of animals) and the breed representation in these datasets was similar and relative to their occurrence in industry flocks (e.g. see Fig. 1). Genotypes on the X chromosome were excluded from the analysis. The imputation of WGS genotypes from 50k was performed in two steps. First, for the animals that did not have real high-density (HD) genotypes (all but 2266 key animals), the 50k genotypes were imputed to HD and then in the second step, all imputed and real HD genotypes were imputed to sequence data using a reference of 726 sequenced animals representing multiple European breeds and crosses (further details in [19]). The numbers of sequenced animals from the Merino and Border Leicester breeds were 124 and 22, respectively.

The total number of variants that passed imputation quality controls and included in this study were: 45,670 SNPs in the 50k panel, 485,617 SNPs in the HD panel and 31,154,082 variants in WGS.

### Selection of significant sequence variants

In the discovery population for each trait, we ran a GWAS using the WGS 31,154,082 imputed genotypes. The GWAS results were used to select the ‘top SNPs’ from those associated with variation in the traits of interest. The “top SNPs” were defined as the most significant SNPs below an association threshold of p-value < 10^−3^ within a 100-kb window. The windows started at the proximal end of each chromosome, then sliding 50 kb along to the next 100 kb window until reaching the end of each chromosome. After selection of the most significant SNP per 100 kb window, one of any pair of these top SNPs in strong LD (with an r^2^ > 0.95) was removed using the PLINK software [20]. Some of the selected SNPs were also removed if their MAF was lower than 0.005 in the reference population for a given trait. The final numbers of top SNPs used for each trait to improve the accuracy of genomic predictions are in Table 1. The small number of top SNPs that overlapped with those on the 50k SNP chip were treated as top SNPs in our genomic prediction models, i.e. they were removed from the 50k subset and retained with the top SNPs when fitting the latter set as a separate component to the 50k. However, these overlapping SNPs were retained in the 50k set when only the 50k genotypes were used for prediction.

The model used in GWAS for each trait using Wombat software [21] is as follows:

1 $${\mathbf{y}}^{*} = {\mathbf{Xb}} + {\mathbf{s}}_{i} \alpha_{i} + {\mathbf{ZQq}} + {\mathbf{Zg}} + {\mathbf{e}},$$

where ${\mathbf{y}}^{*}$ is a vector of corrected phenotypes; ${\mathbf{b}}$ is a vector of fixed effects, intercept and data source, (six industry data sources and one for research) ${\mathbf{s}}_{i}$ is a vector of genotypes (coded 0, 1, and 2), $\alpha_{i}$ is the effect of the $i$th sequence variant; ${\mathbf{q}}$ is a vector with random breed effects, ${\mathbf{q}}\sim {\text{N}}\left( {{\mathbf{0}},{\mathbf{I}}\upsigma_{\text{q}}^{2} } \right)$, ${\mathbf{Q}}$ is a matrix with breed proportion of each animal according to pedigree information, i.e. assigning animals to breeds, and $\upsigma_{\text{q}}^{2}$ is the variance explained by the breed proportion matrix; ${\mathbf{g}}\sim {\text{N}}\left( {{\mathbf{0}},{\mathbf{G}}\upsigma_{\text{g}}^{2} } \right)$, contains random additive genetic effects where ${\mathbf{G}}$ is the genomic relationship matrix (GRM) constructed with HD SNPs and $\upsigma_{\text{g}}^{2}$ is the variance explained by SNPs; ${\mathbf{e}}\sim {\text{N}}\left( {{\mathbf{0}},{\mathbf{I}}\upsigma_{\text{e}}^{2} } \right)$, is the vector of random residual effects; ${\mathbf{X}}$ and ${\mathbf{Z}}$ are design matrices connecting phenotypes to fixed effects and to random additive genetic effects, respectively. The contributions of each breed are listed in Fig. 1, but for the Merino breed three sub-breed groups were defined because there is generally little intercrossing of these sub-breed groups, thus creating further population structure. The Merino sub-breed groups are largely defined on fiber diameter: ultrafine, fine-medium and broad (“strong”) wool, according to wool breeding objectives as defined by the breeders.

### Genomic prediction

We applied genomic BLUP (GBLUP) [22] and the BayesR [23] and BayesRC methods [24] for prediction of genomic breeding values (GBV). The same phenotypic data were used in the Bayesian and GBLUP models, and prior to these analyses, the corrected phenotypes for animals in the training and validation sets were further pre-adjusted for data source and genetic groups using estimates for those effects (${\mathbf{b}}$ and ${\mathbf{q}}$) based on the model in Eq. 1, but the individual SNP effect was not included in the model.

Five different genotype sets were tested in the models: (1) top sequence variants (top), (2) 50k panel, (3) 50k plus top sequence variants (50k + top), and (4) HD panel and 5) WGS data. However, due to the large number of SNPs in WGS data, we did not run a Bayesian model for that set. The SNPs with a minor allele frequency (MAF) lower than 0.005 in the genomic prediction training populations were excluded from the genotype sets. In the GBLUP models, top, 50k, 50k + top, HD and WGS genotype sets were fitted in the model with the GRM as a covariance structure of one random additive genetic effect (Eq. 2). In addition, the 50k + top set was also fitted as two GRM constructed separately from the 50k and top SNP genotype sets (Eq. 3):

2 $${\mathbf{y}} = {\mathbf{1}}\mu + {\mathbf{Zu}}_{1} + {\mathbf{e}},$$

3 $${\mathbf{y}} = {\mathbf{1}}\mu + {\mathbf{Zu}}_{1} + {\mathbf{Zu}}_{2} + {\mathbf{e}},$$

where ${\mathbf{y}}$ is a vector of pre-adjusted phenotypes; ${\mathbf{1}}$ is a vector with 1s, $\mu$ is the intercept; ${\mathbf{Z}}$ is a design matrix allocating records to individual additive genetic values in ${\mathbf{u}}_{1}$ (${\mathbf{u}}_{1} \sim {\text{N}}\left( {{\mathbf{0}},{\mathbf{G}}_{1} \upsigma_{{{\text{g}}1}}^{2} } \right)$), which is a vector of GBV in which ${\mathbf{G}}_{1}$ is the GRM constructed from different sets of genotypes (i.e. top, 50k, 50k + top, HD and WGS) and $\upsigma_{{{\text{g}}1}}^{2}$ is the additive genetic variance; and ${\mathbf{e}}$ is a vector of random residual effects. When 50k + top genotypes were fitted as two components as in Eq. (3), ${\mathbf{u}}_{1} \sim {\text{N}}\left( {{\mathbf{0}},{\mathbf{G}}_{1} \upsigma_{{{\text{g}}1}}^{2} } \right)$ and ${\mathbf{u}}_{2} \sim {\text{N}}\left( {{\mathbf{0}},{\mathbf{G}}_{2} \upsigma_{{{\text{g}}2}}^{2} } \right)$ are vectors of additive genetic values explained by the SNP sets that formed ${\mathbf{G}}_{1}$ and ${\mathbf{G}}_{2}$ from the 50k and top SNP genotype sets, respectively, and $\upsigma_{{{\text{g}}1}}^{2}$ and $\upsigma_{{{\text{g}}2}}^{2}$ are the respective additive genetic variances of those effects. The overall GBV for each individual was formed from the sum of ${\mathbf{u}}_{1}$ and ${\mathbf{u}}_{2}$. We used the MTG2 software [25] for GBLUP of GBV and genomic residual maximum likelihood (GREML) for estimating variance components.

For Bayesian analysis, the genotypes were centered and standardized to a variance of 1. The SNP effects were fitted as a mixture of four normal distributions each with a mean of zero and variance: $\upsigma_{1}^{2} = 0$, $\upsigma_{2}^{2} = 0.0001\upsigma_{\text{g}}^{2}$, $\upsigma_{3}^{2} = 0.001\upsigma_{\text{g}}^{2}$ and $\upsigma_{4}^{2} = 0.01\upsigma_{\text{g}}^{2}$, where $\upsigma_{\text{g}}^{2}$ is the additive genetic variance. The BayesR method was used to predict GBV using, top, 50k, 50k + top and HD genotype sets. The BayesRC method was only tested with 50k + top genotypes because this is a modified BayesR method that allows for different categories of variants that may be differentially enriched for QTL. Thus, the selected top SNPs were allocated to a separate category from the remaining 50k SNP, allowing the possibility of a different mixture distribution of SNP effects in each of these two categories. Each Bayesian model (BayesR and BayesRC) was replicated with five MCMC chains to check for convergence, each with 40,000 iterations (20,000 burn-in). Animal GBV were calculated by multiplying genotypes and corresponding SNP effects summed across the genome. The total proportion of phenotypic variance explained by all SNPs was used to estimate trait heritabilities.

Evaluation of the accuracy of genomic prediction was calculated as the Pearson correlation coefficient between GBV and pre-adjusted phenotypes in the two validation subsets and then scaling this correlation by dividing it by the square root of the trait heritability (estimated in a GBLUP model using a 50k genotype set, Table 2). The bias of the prediction accuracy was assessed from the regression coefficient of the pre-adjusted phenotype on GBV in the validation subsets. The Bayesian heritability, accuracy and bias were assessed from each of the five MCMC chains and then averaged.

**Table 2:** The estimated heritability for different traits in Bayesian and GBLUP models based on the reference dataset

Model	Trait							
	CCFAT	CEMD	PEMD	IMF	SF5	PWT	YCFW	YFD
Bayesian								
BayesR (top)	0.094	0.065	0.129	0.163	0.105	0.112	0.219	0.611
BayesR (50k)	0.196	0.155	0.228	0.376	0.189	0.219	0.393	0.547
BayesR (50k + top)	0.201	0.160	0.242	0.378	0.189	0.224	0.400	0.634
BayesRC (50k + top)	0.190	0.159	0.230	0.359	0.161	0.217	0.384	0.672
BayesR (HD)	0.224	0.185	0.274	0.409	0.222	0.242	0.433	0.596
GBLUP								
GBLUP (top)	0.102	0.070	0.134	0.166	0.108	0.116	0.234	0.619
GBLUP (50k)	0.200	0.150	0.229	0.380	0.189	0.216	0.399	0.569
GBLUP (50k + top [1GRM])	0.217	0.158	0.250	0.400	0.203	0.232	0.409	0.679
GBLUP (50k + top [2GRMs])^a^	0.191 (0.116 + 0.075)	0.152 (0.111 + 0.041)	0.226 (0.128 + 0.098)	0.356 (0.252 + 0.105)	0.142 (0.046 + 0.096)	0.212 (0.152 + 0.060)	0.388 (0.271 + 0.117)	0.681 (0.154 + 0.527)
GBLUP (HD)	0.232	0.182	0.275	0.423	0.233	0.250	0.439	0.620
GBLUP (WGS)	0.231	0.191	0.285	0.439	0.240	0.254	0.453	0.643

*CCFAT* carcass fat depth at C site, *CEMD* carcass eye muscle depth, *PEMD* post-weaning eye muscle depth, *IMF* intermuscular fat percentage, *SF5* shear force measured at day 5 after slaughter, *PWT* post-weaning weight, *YCFW* yearling clean fleece weight, *YFD* yearling fiber diameter

^a^The genetic variance explained by two GRM fitted in the model were divided to the phenotypic variance and then were added up to calculate the overal heritability. The first and the second value in the parentheses are the heritability estimates related to 50k and top SNPs, respectively

## Results

### Variance components and heritability estimates

Table 2 shows the heritability estimates of each trait in the prediction data for each of the five SNP subsets when using one of the Bayesian methods or genomic residual maximum likelihood (GREML) with either one or two GRM. Across all traits and SNP subsets, heritabilities estimated based on the Bayesian method were slightly lower (1.6% in relative terms) than those based on the GREML method using a GBLUP model. The heritability estimated by GREML, on average across traits, increased by ~ 15% when using all WGS variants compared to 50k genotypes, but the increase was ~ 25% for CEMD, PEMD and SF5. Heritability estimates based on only selected sequence variants (3924 to 8654 variants) were more than 40% lower than those based on the 50k set, except for YFD where the top SNPs resulted in a higher heritability than the 50k set for both GREML and BayesR. The heritability based on fitting 50k genotypes and selected sequence variants as one component was on average slightly higher than the heritability estimate based on the sum of the two variance components estimated from the 50k and top SNPs sets. Again, YFD was an exception as it always gave a significantly higher heritability when top-SNPs were used.

### Genomic prediction

**Fig. 2:**
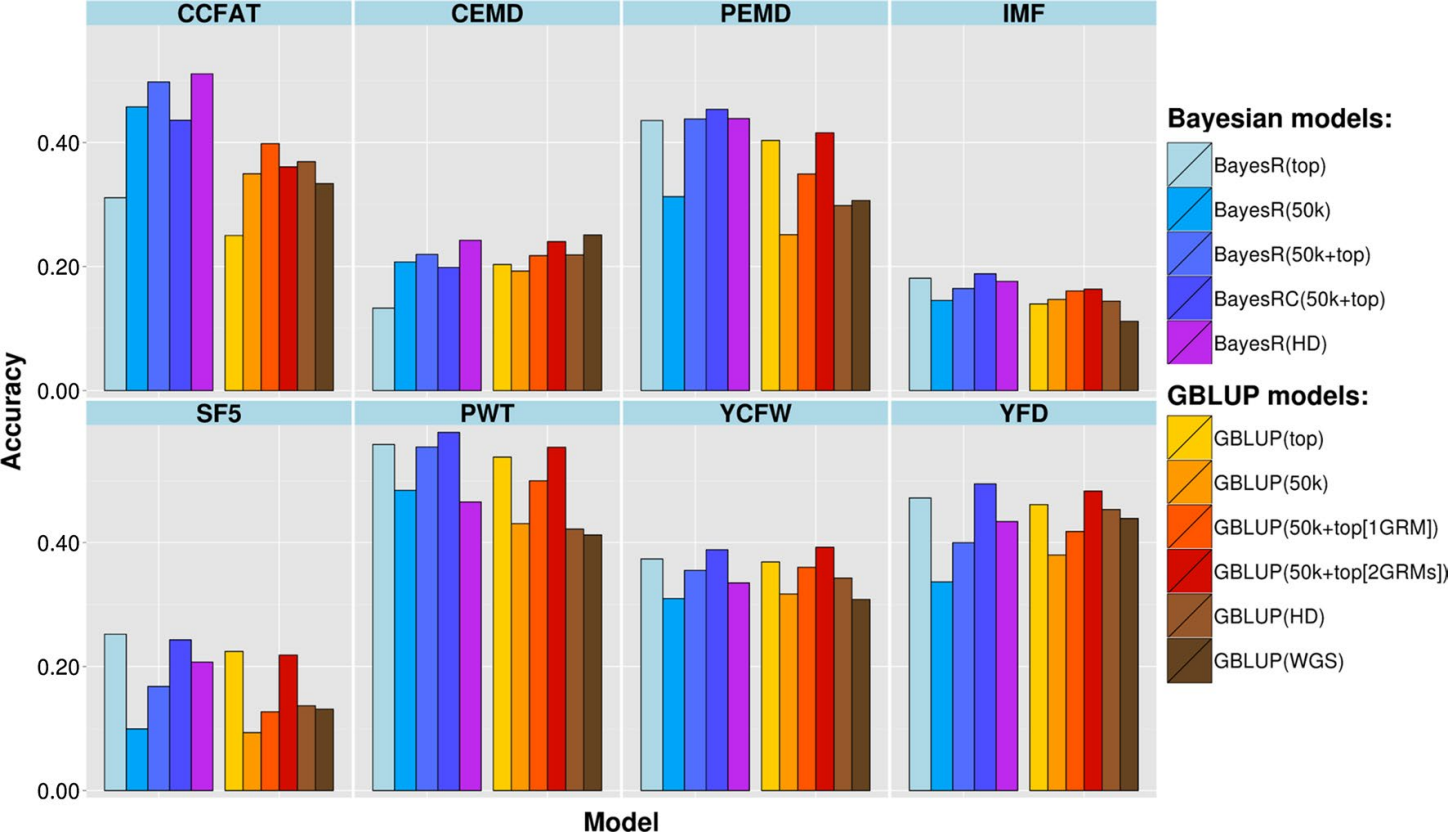
Accuracy of genomic predictions in purebred Merino for different traits and models

**Fig. 3:**
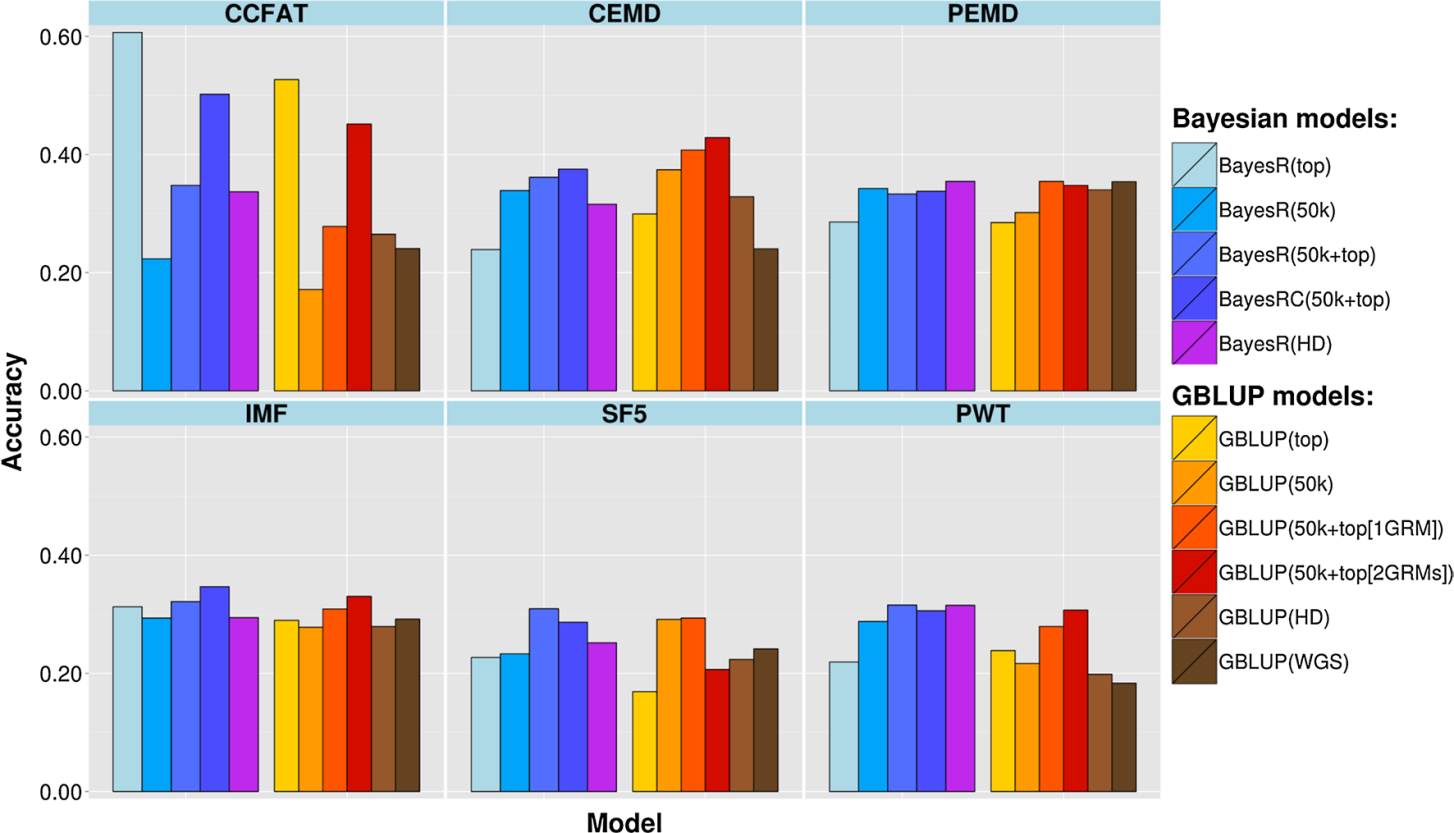
Accuracy of genomic predictions in Merino × Border Leicester crossbreds for different traits and models

The accuracy of genomic prediction for each trait based on different SNP subsets and models is shown in Fig. 2 for the purebred Merino validation set and in Fig. 3 for the crossbred validation set. Currently in the Australian sheep industry, genetic evaluation is based on GBLUP using a 50k SNP array, so we used the scenario GBLUP (50k) here as the benchmark. The average GBLUP (50k) accuracy across all traits was 0.27 for both validation sets, but it should be noted that the validation sets were selected to minimize relationships with the training set (as a more stringent test to differentiate between methods).

Accuracy of GBV based on the HD SNP genotypes and GBLUP was only slightly increased relative to GBLUP (50k) (0.028 accuracy increase for Merinos and no difference for the crossbreds when averaged across traits), but increases were higher and more consistent with BayesR (0.081 for Merinos and 0.039 for the crossbreds). When using all the variants in WGS data (~ 31 × 10^6^ variants) the accuracy of genomic prediction increased in some cases compared to either the 50k or HD GBLUP, but more often decreased with no clear pattern among traits. Compared to using 50k, the WGS set resulted in a change in accuracy, averaged across traits, of + 0.016 for Merinos and − 0.014 for the crossbreds.

A larger and more consistent increase in prediction accuracy was observed when adding the selected sequence variants to the standard 50k set in the Bayesian and GBLUP prediction models. When using the GBLUP method and fitting selected sequence variants and 50k genotypes as one variance component, the average prediction accuracy across traits improved in absolute value by 0.046 and 0.048 for Merinos and crossbreds, respectively, and these improvements were 0.083 and 0.073 when two separate variance components were fitted (one for 50K and one for the top SNPs). When including top SNPs using the BayesR method, the accuracy increase, when averaged across traits, was 0.079 for Merinos and 0.059 for crossbreds, compared with GBLUP (50k), and these increases were 0.102 and 0.087 with the BayesRC method, in which top SNPs and the 50k set were treated, separately.

**Fig. 4:**
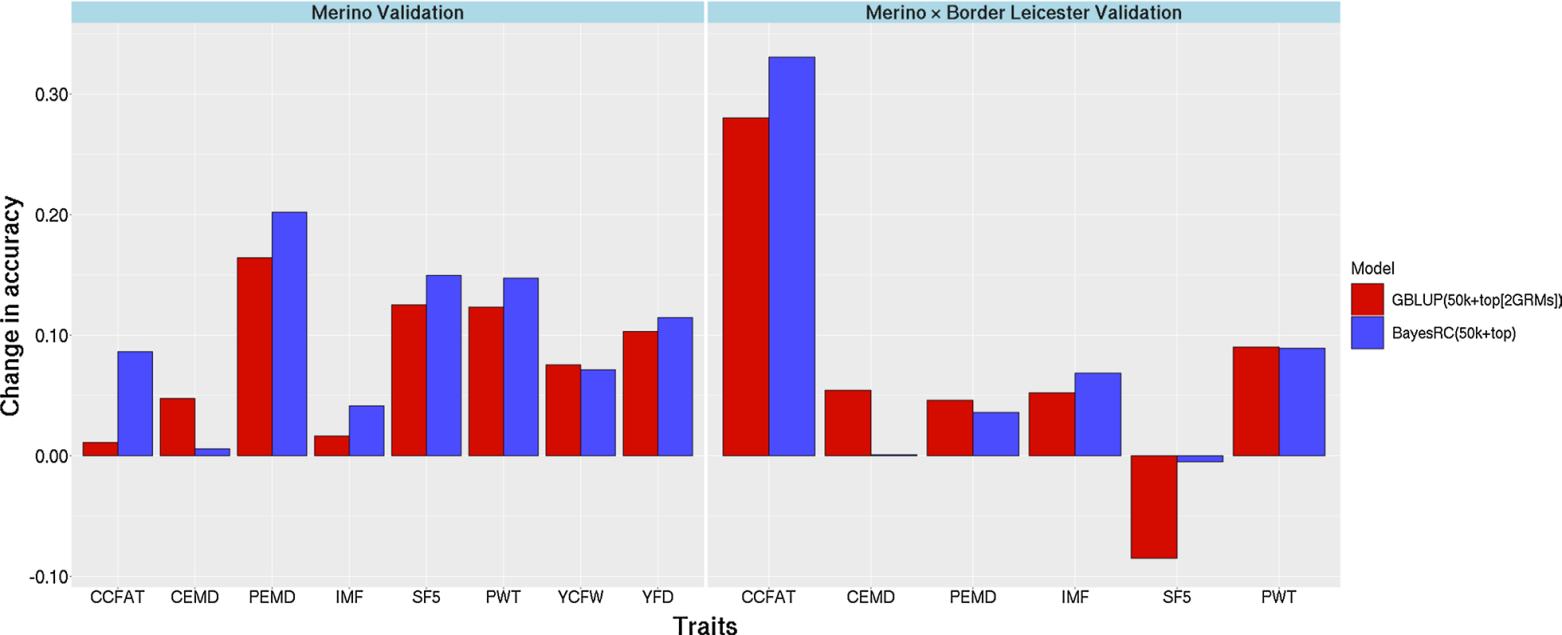
Accuracy increase for each trait when using top SNPs in GBLUP or BayesRC. Accuracy increase over a 50k GBLUP prediction for each of the traits in both validation populations when using top SNPs either as a second GRM in GBLUP or as a second class of predictors in BayesRC

The increase in accuracy due to adding top SNPs to the 50k array varied among traits, between methods within traits, and also with the validation set (Fig. 4). With GBLUP in the Merino set, the increase from using top SNPs as an additional ${\mathbf{G}}$ matrix compared to using the standard 50k was lowest for CCFAT (0.01) and IMF (0.02), and highest for PEMD (0.16), SF5 (0.13) and PWT (0.12). When using BayesRC, the increases of using the top SNPs added to the standard 50k array, compared to using GBLUP (50k), were 0.09, 0.04, 0.20, 0.15 and 0.15 for the same traits in the Merino set. However, in crossbreds, these increases were quite different across traits. For both GBLUP and Bayesian prediction methods, most traits had an increase in accuracy of ~ 0.05 when adding information from the top SNPs, but CCFAT had a much higher increase of 0.28.

Using just the set of selected variants alone improved accuracy more compared with GBLUP (50k) when averaged across all traits; 0.053 and 0.029 for GBLUP in Merino and crossbred validation sets and 0.069 and 0.043 for BayesR in these two validation sets (Figs. 2 and 3). However, there was again a large variation among traits and validation populations, which was generally consistent with the increase in accuracy when combining top SNPs with 50k panels. For example, in the Merino set, top SNPs alone had a much higher accuracy than GBLUP (50k) for PEMD and SF5 whereas for CCFAT and CEMD this accuracy was lower than the 50k array. On the other hand, for the crossbred validation population, CCFAT showed a much higher accuracy when using top SNPs only (increase 0.36) compared to using GBLUP (50k), whereas for the other traits there was either a lower accuracy or only a slightly higher accuracy than that of the 50k panel.

**Fig. 5:**
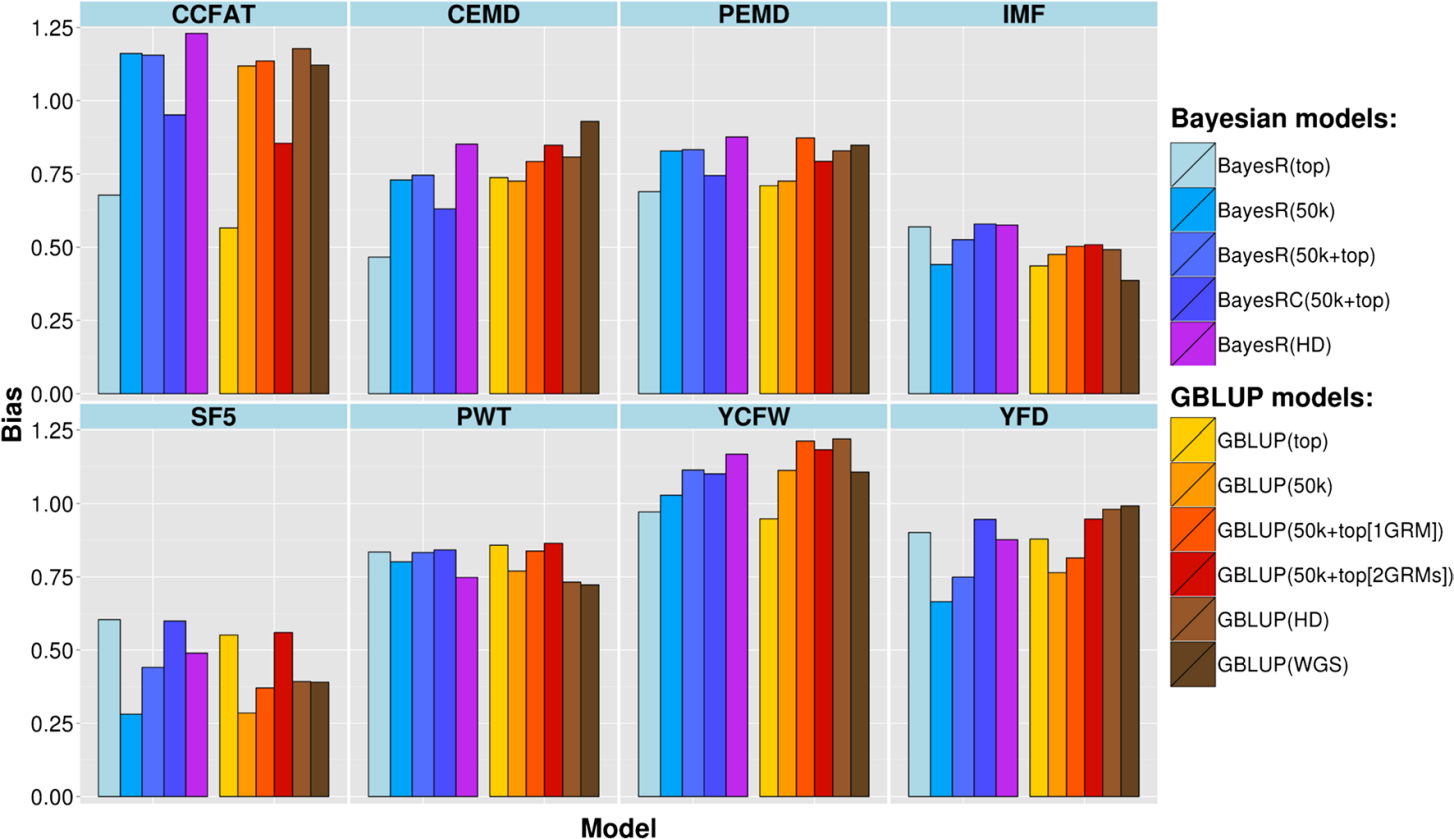
Bias of genomic predictions in purebred Merino for different traits and models

**Fig. 6:**
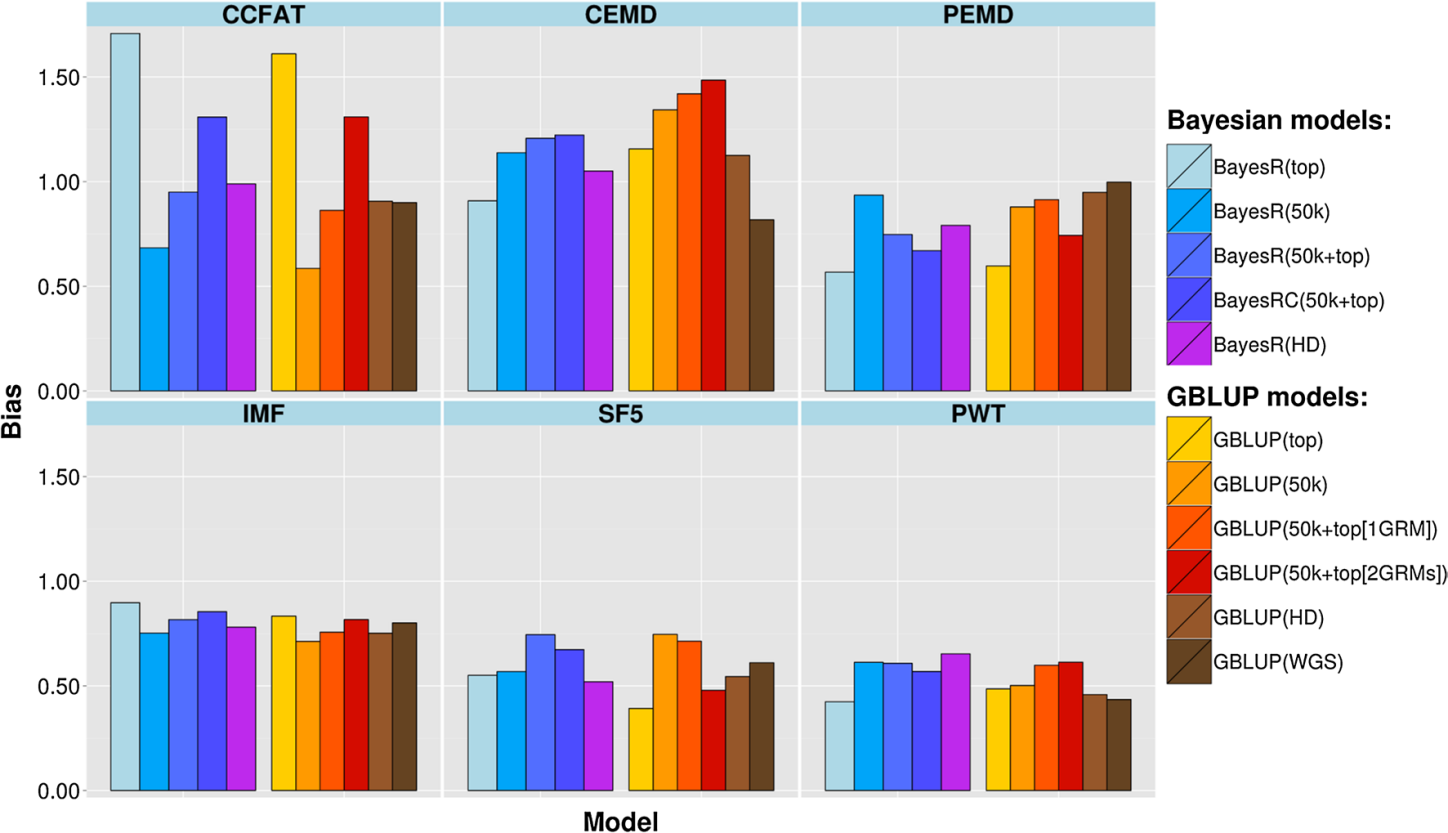
Bias of genomic predictions in Merino × Border Leicester crossbreds for different traits and models

The regression coefficients of corrected phenotypes on estimated GBV for two validation sets across different traits are shown in Figs. 5 and 6. All methods and models showed some bias. Regression coefficients, when averaged across traits, varied between methods from 0.71 to 0.85 for the Merinos and from 0.76 to 0.91 for the crossbred validation population. In the Merino set, the greatest deviation from 1 in the average regression coefficient (i.e. most bias) was found when using top SNPs alone (0.71), but this was only slightly lower than the average value for GBLUP (50k), which was 0.74. For the crossbreds, on average bias was not larger when top SNPs were used, but this average was affected by a strong upward bias for one trait (CCFAT). There was no difference in bias between GBLUP and BayesR. Generally, we found more bias for traits with lower prediction accuracy. This could be related to the smaller size of both training and validation data subsets for those traits.

## Discussion

This study demonstrated that the use of selected variants from WGS increased the accuracy of genomic prediction compared to standard 50k genotypes for eight economically important traits in sheep. Selection of the WGS variants (SNPs or InDels) was based on performing an association study on imputed sequence data in an independent set of animals to avoid probable bias of prediction [11, 13]. The accuracy of genomic prediction was assessed in independent training and validation datasets, chosen in such a way as to remove genetically high relationships between the validation and training animals. Thus, genomic prediction accuracies are expected to be lower and the benefit from using selected sequence variants is likely higher, e.g. compared with dairy cattle studies where populations are less diverse and training populations often contain highly related individuals. However, in beef and sheep populations, individual breed numbers are small and therefore it is desirable to combine multi-breed and crossbred populations to enlarge reference populations for genomic prediction. Therefore, in these more genetically diverse beef cattle or sheep populations, the prediction of more distant relatives is also important. The same holds for genomic prediction of commercial animals, e.g. for the purpose of genetic bench-marking and management decisions related to genetic merit. Furthermore, there is a trend towards highly accelerated breeding schemes using reproductive technologies, where selection candidates may be separated from the reference population by several generations. It is expected that genomic prediction based on predictive markers that are closer to actual causal variants are more widely applicable and depend less on the relatedness between the training set and the target individual. This is also important for difficult to measure traits for which it may not be possible to continually update reference populations with immediate relatives of test animals.

Compared to using 50k genotypes, using the complete set of sequence data (~ 31 million variants) resulted in a higher estimated additive genetic variance and heritability. We compared the sum of diagonal terms and off-diagonal terms from ${\mathbf{G}}$-matrices derived from 50k, HD and WGS genotypes and both means and SD were nearly identical between these. Therefore, a higher heritability estimate could potentially be the result of stronger LD between sequence variants and polymorphisms responsible for trait variation. However, no or only a very small improvement in genomic prediction accuracy was observed when using all sequence variants compared to 50k genotypes. This finding is similar to previous studies in dairy cattle [7–9, 11, 12] that also found no to very small improvements in prediction accuracy from using all variants of WGS. WGS data provide a very large number of genetic variants across the genome, while only a small subset is expected to be, or in high LD with, the causative mutations underlying the genetic variance of a polygenic trait. This means that the majority of the sequence variants are in low LD with causative mutations and do not capture the genetic variance of significant genomic regions when using a GBLUP approach. Thus, compared to the 50K genotypes, their contribution would be limited to the capture of more precise family relationships between animals. It is possible that the prediction accuracy for WGS showed little benefit in lowly related validation individuals because a large proportion of the markers are not linked to causal variants in the validation set and might hinder rather than help prediction accuracy. Furthermore, in WGS there is a much higher risk of false positives from for example, population structure, as well as imputation errors which can erode the accuracy of genomic prediction [9].

Estimation of heritability is mainly driven by (higher) relationships between training animals, in which case markers that are not linked to causal variants could still be helpful in estimating these more accurately. This argument can also be used to explain that the heritability based on using top SNPs alone was generally lower than the heritability based on the SNP sets with more markers, while prediction accuracies based on these selected marker sets were generally similar and sometimes even higher than the accuracy based on the 50k set. A similar result was found by Raymond et al. [26] who argue that the selected variants may explain only a small amount of the genetic variation due to their small effects, but they can contribute significantly to genomic prediction when relationships between training and target individuals are low, in their case across breed. Besides marker density and potential proximity of markers to causal variants, another potential factor that may influence estimates of heritability across variant sets could be their difference in allele frequency spectrum.

Fitting the selected sequence variants (3924 to 8654 for different traits) that were derived from the association study along with the standard 50k genotypes often resulted in a notable increase in prediction accuracy in both purebred and crossbred animals, particularly if these selected markers were fitted as separately defined groups, either in GBLUP (2 ${\mathbf{G}}$ matrices) or in BayesRC. These methods allow more variance for the set of selected SNPs and therefore less shrinkage and effectively giving them more weight in the prediction. We expected an increase because these selected genetic variants are in stronger LD with QTL regions than randomly chosen markers such as those on the 50k array. However, as shown in Fig. 4, the gain in accuracy varied among traits and validation sets. Variation between traits could exist due to the differences in the genetic architecture of each trait, i.e. there would be more benefit from using top SNPs if a trait is affected by relatively few QTL, each with a relatively large effect. However, our results varied also within traits and among validation sets, e.g. selected variants for CCFAT explained a small part of the genetic variation in the Merino validation set and had only a small effect on prediction accuracy, while these two parameters were much larger in the crossbred validation set. This could be related to differences in QTL effects between populations that could be due to epistatic interactions, or simply due to differences in mean and variation of traits in different breeds. Allele frequency differences between breeds are likely to have a large impact on results, both for QTL and predictive markers. We examined furthermore the high accuracy and bias for CCFAT found in the crossbred validation when only fitting top SNPs. We noted that these results were quite sensitive to the MAF threshold applied, i.e. both accuracy and bias were lower in this case when the MAF threshold for the top SNPs to be included increased from 0.005 to 0.05. Evans et al. [27] recently demonstrated that low MAF WGS variants can have a considerable effect on the estimation of SNP heritability. Further work is needed to systematically explore all these issues with larger datasets.

Besides finding a set of more predictive SNPs, it is also important to examine how these are optimally used in the genomic prediction model. Our study is the first to compare directly the single and two GRM GBLUP models vs. BayesR and BayesRC methods. The GBLUP linear model with two separate variance components allows for a larger genetic variance on average per marker for the set of selected SNPs, effectively giving more weight to these markers. In principle, more ${\mathbf{G}}$-matrices could be fitted to allow further differential weighting of markers into more subclasses, where marker selection would be based on statistical significance in a separate GWA analysis. Alternatively, it is possible to use a single ${\mathbf{G}}$ matrix that is weighted, for example, according to the estimated predictive value of each SNP from one or more independent studies [28]. This two-step approach is pragmatic and feasible in large datasets and with very many markers, such as from WGS data. A Bayesian model such as BayesR applies the concept of differentially weighing markers more formally, by assigning marker effects to one of the four normal distributions based on statistical evidence in a model selection approach in which all markers are fitted simultaneously, and allowing for a large proportion of markers to have no effect on the trait. The BayesRC model takes the standard BayesR model one step further by enabling the mixing proportions to vary across different variant sets, where based on prior information, the user defines one or more groups of variants that may be more enriched for QTL than another. In this study, the variant sets were the 50k and the top SNP set, and for many traits, this improved prediction accuracy over BayesR. However, although approaches for Bayesian analysis with the complete set of WGS markers have been developed [8, 9], these models did not result in increased accuracy for WGS compared to standard genotype sets using real dairy cattle data. Van den Berg et al. [14] found limited benefit over 2GBLUP from a Bayesian model fitting two mixture distributions. Our design was more favorable towards showing benefit of using selected variants, and the BayesR methods showed more clearly an advantage over GBLUP methods. However, Bayesian methods also become very computationally challenging in larger datasets with hundreds of thousands of individuals and dense genotype sets. However, large datasets are needed to obtain more accurate estimates of marker effects and to enable more accurate marker selection. Further development of model selection approaches is required, likely with a role for two-stage approaches, to optimize the selection of WGS variants and their best weighting for the purpose of genomic prediction of trait variation.

Our results were based on a multi-breed dataset and, generally, the improvement in prediction accuracy from using selected variants was larger than that obtained in studies using data on a single breed (mostly Holstein–Friesian dairy cattle e.g. [7, 11, 12]. This is in line with other studies in dairy cattle, which used multi-breed datasets [10, 14]. Van den Berg et al. [14] used variants derived from multi-breed GWAS (four dairy cattle breeds) in multi-breed genomic prediction and showed on average up to 7% higher genomic prediction reliabilities (*R*^2^) across milk traits in different scenarios for selecting sequence variants. By including selected sequence variants from GWAS in GBLUP (with a separate variance component to the 50k genotypes) Brøndum et al. [10] reported on average a 5% improvement in genomic prediction reliability on a range of production traits in a multi-breed dairy cattle dataset. Multi-breed datasets are particularly useful in GWAS for marker selection because long distance LD will be reduced across breeds, allowing a more precise localization of predictive markers across breeds. We have not tested explicitly our ability to increase prediction accuracy from training data on different breeds. The accuracy of prediction across breeds (i.e. the reference and the validation sets consist of individuals from different breeds) was found to be close to zero in our previous work with less dense marker panels [4, 5]. Some results from data in dairy cows showed promise for the use of information from other breeds and this might be mostly important for smaller breeds that often lack large numbers for training genomic predictions [29, 30].

Using multi-breed data for marker selection could also have a drawback if marker effects differ between populations. In our data, the number of purebred individuals from different breeds was not sufficient to test this hypothesis. In our validation sets, we observed on average a similar prediction accuracy for purebred Merinos and for BL × Mer crosses, but the benefit of using selected variants was generally higher in the Merino validation set. This is likely due to the larger proportion of Merino haplotypes that were available in the training and SNP-discovery sets. Generally, Merino sheep are a lot more diverse than BL sheep and previous work has shown that a smaller training set for BL results in a similar accuracy to that for Merinos based on a large training set [5]. In other words, Merinos have a large effective population size, and as a result, a larger number of effective chromosome segments. This would also explain that the use of selected markers that are in higher LD with QTL is more advantageous for the Merino breed than the BL breed, which has less diversity and a larger effective number of chromosome segments.

We used a design in which the discovery set used for GWAS and SNP selection was separate from the training set used to estimate marker effects for the purpose of genomic prediction. Previous papers pointed out that using the same data for discovery and training leads to biased predictions [11, 13]. Our results show that prediction based on top SNPs only were slightly more biased than prediction without these selected sets. This may be due to selected variants picking up some effects of the population structure that were not accounted for by the genetic groups that were derived from pedigree. Population structure is pronounced in the Merino breed where various subpopulations exist that are quite distinct, and therefore more precise correction for population structure based on genomic relationships might have resulted in less bias. Differences in bias between using selected variants and all variants was found to be much larger in a single-breed study with Holstein–Friesian cattle [11], where QTL discovery and genomic prediction training sets were the same.

We used an arbitrary proportion of the total dataset for SNP discovery. A balance needs to be found between the accuracy of selecting top SNPs and the precision of establishing the accuracy of prediction. The process of allocating data to discovery, training and validation sets could also be repeated multiple times, so that results can be averaged over multiple samples. For larger datasets, a larger proportion of the data could be used perhaps for discovery. It is tempting to use the complete dataset to accurately establish QTL and markers to predict their effects. However, it would then be impossible to establish the improvement in prediction accuracy free of bias. In addition, in commercial genetic evaluations, where top SNPs are selected from the same data as those used for prediction, it is likely that genomic predictions of breeding values are biased. Bias could be the result of selectively using information from random occurrences, but also systematic biases could occur due to not accounting properly for population structure, or otherwise use of selective data. The extent of the problem may be smaller with larger datasets but some cautions and independent validation will be required. The ultimate goal is to generate custom SNP panels for genotyping that include highly predictive sequence SNPs for the entire range of economically important traits, so that these variants can be directly genotyped rather than rely on imputation to WGS that is more error prone.

The threshold for the p-value of marker effects in the selection of sequence variants to be used in genomic prediction was set at p < 10^−3^. Other studies have varied the threshold and, generally, found no or a slight improvement if more stringent thresholds were used [11, 14, 31]. An optimal threshold is part of the process of selecting variants to improve genomic prediction, and an optimal value might well differ between traits and datasets. For example, a more stringent threshold is easier to apply in large datasets, since the false discovery rate can be low without losing too much power. An important aspect of our approach was the relatively lenient p-value threshold combined with selection of only one “top SNP” per 100-kb window along the entire genome. This enables representation of many of the QTL with a smaller effect that would otherwise be missed and reduces redundancy of multiple markers tagging a single QTL.

This paper describes the first study in sheep to test the selection of WGS variants for improving prediction accuracy. Compared with previous studies, results were relatively more consistent with larger accuracy increases compared with those described in studies on dairy cattle. This may be partly due to the large diversity and multi-breed nature of the data used in sheep breeding. However, a large part of the additional contribution is also likely the result of the lower accuracy overall, as achieved for the default scenario of 50k industry panel. Our training dataset is smaller than those used in most dairy studies, and we have tested the accuracy in relatively unrelated animals, which is more difficult to achieve in populations that are less diverse. In the near future, we can expect that larger datasets will become available, which will be favorable towards more accurate SNP selection. Larger training datasets will of course also increase the prediction accuracy overall, even with standard SNP arrays. However, adding selected variants from WGS data to SNP arrays used in the industry will have a substantial benefit, especially for predicting GBV of animals that are not well connected to the industry reference population, provided suitable analytical methods are applied.

## Conclusions

Use of selected variants from imputed whole-genome sequence data resulted in considerable improvement in genomic prediction accuracy in validation sets that had a relatively low relationship to individuals in the training set. These variants were selected from independent discovery sets and larger increases in prediction accuracy were observed if they were given more weight in the prediction model, either as a separate variance component in GBLUP, or by allocating them to a separate group when using a Bayesian approach that allows for different mixture models of SNP effects (BayesRC).

## Supplementary information

**Additional file 1: Table S1.** Summary statistics of phenotypes for different traits. Table with summary statistics for each trait in the study.
