# Supplementary material for: Genomic prediction based on selected variants from imputed whole-genome sequence data in Australian sheep populations
Source: Genet Sel Evol. 2019 Dec 5;51:72. doi: 10.1186/s12711-019-0514-2 (PMC6896509; doi:10.1186/s12711-019-0514-2)
Supplement: Supplementary file 1 — Additional file 1: Table S1. Summary statistics of phenotypes for different traits. Table with summary statistics for each trait in the study. [file 12711_2019_514_MOESM1_ESM.docx]

**Table S1.** Summary statistics of phenotypes for different traits.

| Trait | Total Number  of records | Phenotypic  Average | Phenotypic SD |
| --- | --- | --- | --- |
| CCFAT (mm) | 13,769 | 4.06 | 2.27 |
| CEMD (mm) | 13,896 | 29.63 | 4.79 |
| PEMD (mm) | 21,240 | 26.13 | 4.64 |
| IMF (%) | 11,872 | 4.45 | 1.17 |
| SF5 (N) | 13,475 | 26.79 | 14.09 |
| PWT (kg) | 26,657 | 46.44 | 12.83 |
| YCFW (kg) | 9,626 | 2.24 | 0.81 |
| YFD (μm) | 10,798 | 17.33 | 1.55 |
